# Vitamin D receptor *Fok*I polymorphism and the risks of colorectal cancer, inflammatory bowel disease, and colorectal adenoma

**DOI:** 10.1038/s41598-018-31244-5

**Published:** 2018-08-27

**Authors:** Young Ae Cho, Jeonghee Lee, Jae Hwan Oh, Hee Jin Chang, Dae Kyung Sohn, Aesun Shin, Jeongseon Kim

**Affiliations:** 1 Department of Cancer Biomedical Science, Graduate School of Cancer Science and Policy, National Cancer Center, Goyang, South Korea; 2 Center for Colorectal Cancer, National Cancer Center Hospital, National Cancer Center, Goyang, South Korea; 3 Department of Preventive Medicine, Seoul National University College of Medicine, Seoul, South Korea

## Abstract

Based on an inverse association between vitamin D levels and the risks of colorectal diseases, a functional start codon polymorphism in the vitamin D receptor (*VDR*) gene is speculated to affect the risks for these diseases. To validate this hypothesis, we first conducted a case-control study of 695 colorectal cancer patients and 1,397 controls. The association of *VDR Fok*I polymorphism with colorectal cancer risk was analyzed using a logistic regression model. In the present case-control study, compared to the F allele, the f allele seemed to be associated with lower risks of colon cancer and advanced colorectal cancer. Additionally, a meta-analysis of 27 studies was conducted to combine findings from previous studies investigating the association of *Fok*I polymorphism with colorectal disease using a random effects model. In the present meta-analysis, the f allele was positively associated with the risk of inflammatory bowel disease, including Crohn’s disease and ulcerative colitis. However, this allele was inversely associated with colon cancer and was not associated with the risk of rectal cancer or colorectal adenoma. In conclusion, the findings from this study imply that the role of *VDR Fok*I polymorphism may differ based on the type and severity of colorectal disease.

## Introduction

Epidemiologic studies have reported that low serum vitamin D levels are associated with an increased risk of colorectal diseases such as colorectal cancer^1^, colorectal adenoma^2^, and inflammatory bowel disease^3,4^. The presence of colorectal adenoma and inflammatory bowel disease may increase the risk of colorectal cancer. Approximately 85% of colorectal cancer cases are thought to evolve from conventional adenomas through a process known as the adenoma-carcinoma sequence^5^. In addition, patients with inflammatory bowel disease have an increased risk of colorectal cancer compared to the general population^6^.

The active form of vitamin D, 1,25(OH)_2_D_3_, exerts its biological functions via the vitamin D receptor (VDR). Therefore, the protective effects of vitamin D against colonic carcinogenesis are most likely mediated through the VDR, which is expressed in approximately 30 different tissues. After binding to 1,25(OH)_2_D_3_, the VDR transactivates genes that modulate the immune response, inhibit proliferation, or promote differentiation and apoptosis^7,8^. Therefore, the associations of *VDR* polymorphisms with the risks of various diseases, including cancer and immune disease, have been extensively examined in epidemiologic studies. Among numerous polymorphisms identified in the *VDR* gene, the *Fok*I (rs2228570) polymorphism is considered an independent marker because it has no linkage disequilibrium with any other *VDR* polymorphisms^9^. Additionally, this polymorphism changes the first start codon in the gene from ATG to ACG, resulting in a VDR protein that is shorter by three amino acids. The protein encoded by the *Fok*I F allele (C allele) more efficiently binds vitamin D than the longer version coded by the f allele (T allele)^9,10^. Therefore, the f allele may mimic the cellular consequences of lower vitamin D levels.

Previous studies have reported inconsistent findings regarding the associations of *VDR Fok*I polymorphism with the risks of colorectal cancer, colorectal adenoma, and inflammatory bowel disease. Positive, negative, or no associations with these colorectal diseases have been reported^11–14^. Therefore, we hypothesized that the role of this polymorphism may differ by disease status. Since achieving sufficient statistical power to detect associations between polymorphisms and disease risks can be difficult in individual studies, a meta-analysis combining data from all published studies may detect genetic associations more accurately and reduce the probability of false-negative results^15^. Some meta-analyses have discussed the roles of *Fok*I polymorphism^16,17^, but to the best of our knowledge, none have investigated its role in colorectal disease comprehensively.

The aim of this study was to investigate the association between *VDR Fok*I polymorphism and the risk of colorectal disease. Therefore, a systematic meta-analysis was performed to combine data from previous studies on the association between *VDR Fok*I polymorphism and the risks of colorectal diseases, including colorectal cancer, colorectal adenoma, and inflammatory bowel disease. To add more evidence, we also conducted a case-control study in Korea.

## Results

### Findings from the Present Case-Control Study

Compared to the controls, the cases were more likely to have a family history of colorectal cancer (*P* < 0.001), to be less educated (*P* < 0.001), to have a low level of regular exercise (*P* < 0.001), and to have a higher total caloric intake (*P* < 0.001). In contrast, no significant differences were identified between the cases and controls in terms of body mass index (BMI), smoking status, and alcohol consumption (Supplementary Table S1).

*VDR Fok*I polymorphism was not associated with colorectal cancer. However, in a stratified analysis by anatomic location and cancer stage, *Fok*I polymorphism was associated with colon cancer and advanced-stage colorectal cancer at a *P*-value level of 0.05 (Table 1). An association with a lower risk of colon cancer was observed among those carrying the *Fok*I f allele (odds ratio (OR) = 0.75, 95% confidence interval (CI) = 0.57–0.99, *P* = 0.045 for Ff vs. FF; OR = 0.75, 95% CI = 0.58–0.98, *P* = 0.033 for Ff + ff vs. FF); however, this association was not observed for rectal cancer. In addition, an association with a lower risk of colorectal cancer was observed for patients with advanced-stage colorectal cancer harboring the *Fok*I f allele (OR = 0.76, 95% CI = 0.58–0.99, *P* = 0.042 for Ff vs. FF; OR = 0.77, 95% CI = 0.60–1.00, *P* = 0.047 for Ff + ff vs. FF).

**Table 1:** Association between *VDR Fok*I polymorphism and the risk of colorectal cancer in the case-control study.

*VDR Fok*I	Number (%)		Crude OR (95% CI)	*P*-Value	Adjusted OR (95% CI)^(a)^	*P*-value
	Controls	Cases				
Colorectal cancer						
FF	448 (32.1)	252 (36.3)	1.0 (ref)		1.0 (ref)	
Ff	697 (49.9)	315 (45.3)	0.80 (0.66–0.99)	0.035	0.83 (0.66–1.04)	0.104
ff	252 (18.0)	128 (18.4)	0.90 (0.69–1.17)	0.447	0.92 (0.68–1.24)	0.591
Ff + ff vs. FF			0.83 (0.69–1.01)	0.056	0.85 (0.69–1.06)	0.145
f allele vs. F allele			0.93 (0.81–1.05)	0.240	0.94 (0.81–1.09)	0.386
**Tumor location**						
Colon cancer						
FF	448 (32.1)	137 (38.9)	1.0 (ref)		1.0 (ref)	
Ff	697 (49.9)	157 (44.6)	0.74 (0.57–0.95)	0.020	0.75 (0.57–0.99)	0.045
ff	252 (18.0)	58 (16.5)	0.75 (0.53–1.06)	0.105	0.75 (0.52–1.09)	0.134
Ff + ff vs. FF			0.74 (0.58–0.94)	0.015	0.75 (0.58–0.98)	0.033
f vs. F			0.84 (0.71–0.99)	0.044	0.84 (0.70–1.01)	0.067
Rectal cancer						
FF	448 (32.1)	111 (33.6)	1.0 (ref)		1.0 (ref)	
Ff	697 (49.9)	138 (46.4)	0.89 (0.68–1.16)	0.382	0.93 (0.69–1.25)	0.624
ff	252 (18.0)	66 (20.0)	1.06 (0.75–1.49)	0.750	1.12 (0.77–1.62)	0.565
Ff + ff vs. FF			0.93 (0.72–1.20)	0.584	0.98 (0.74–1.29)	0.875
f vs. F			1.01 (0.85–1.20)	0.927	1.04 (0.86–1.25)	0.688
**AJCC stage**						
Stage 0 + I + II						
FF	448 (32.1)	74 (32.6)	1.0 (ref)		1.0 (ref)	
Ff	697 (49.9)	107 (47.1)	0.93 (0.68–1.28)	0.653	0.87 (0.61–1.23)	0.426
ff	252 (18.0)	46 (20.3)	1.11 (0.74–1.65)	0.624	1.05 (0.68–1.62)	0.834
Ff + ff vs. FF			0.98 (0.72–1.32)	0.874	0.92 (0.66–1.27)	0.597
f vs. F			1.04 (0.85–1.26)	0.735	1.00 (0.81–1.25)	0.981
Stage III + IV						
FF	448 (32.1)	165 (38.3)	1.0 (ref)		1.0 (ref)	
Ff	697 (49.9)	189 (43.9)	0.74 (0.58–0.94)	0.013	0.76 (0.58–0.99)	0.042
ff	252 (18.0)	77 (17.9)	0.83 (0.61–1.13)	0.240	0.82 (0.58–1.17)	0.275
Ff + ff vs. FF			0.76 (0.61–0.95)	0.017	0.77 (0.60–1.00)	0.047
f vs. F			0.88 (0.75–1.02)	0.097	0.88 (0.74–1.04)	0.141

^(a)^Adjusted for age, sex, BMI, education, family history of colorectal cancer, regular exercise, and total caloric intake. Abbreviations: AJCC, American Joint Committee on Cancer; CI, confidence interval; OR, odds ratio; VDR, vitamin D receptor.

### Findings from the Meta-Analysis

Using our search criteria, a total of 101 articles were retrieved. After evaluating these articles, 76 were excluded for several reasons and 1 was added after screening the references of the retrieved publications and review papers. Therefore, we identified a total of 26 articles on the association between *VDR Fok*I polymorphism and colorectal diseases and included the results from the current case-control study. Finally, 27 studies investigating various colorectal diseases were included in this meta-analysis: colorectal cancer (16 studies; 10,257 cases/12,492 controls)^11,12,18–30^, colorectal adenoma (4 studies; 1,322 cases/1,420 controls)^14,31–33^, ulcerative colitis (6 studies; 1,555 cases/2,295 controls)^13,34–38^, and Crohn’s disease (4 studies; 946 cases/1,390 controls)^34–36,39^. Three articles investigated both ulcerative colitis and Crohn’s disease^34–36^. A study flowchart depicting the literature search and selection process is presented in Supplementary Fig. S1. Table 2 presents the characteristics of the studies included in the meta-analysis.

**Table 2:** Characteristics of the studies included in the meta-analysis.

First author (year)^ref^	Country	Ethnicity	Disease	Control source	Cases/Controls			MAF	Quality Assessment
					Number	Age (year)	Female (%)		
Simmons (2000)^34^	UK	Caucasian	UC, CD	P	323/101	NR	58/NR	0.39	7
Ingles (2001)^14^	USA	Caucasian, African, Hispanic, Asian	CA	P	373/394	62.3/62.2	32/34	0.37	10
Peters (2001)^31^	USA	Caucasian, other	CA	H	208/184	60^b^/57^b^	23/37	0.33	9
Wong (2003)^18^	Singapore	Asian^a^	CRC	P	217/890	66/56.5	42/57	0.47	8
Murtaugh (2006)^11^	USA	Caucasian, Hispanic, African	CRC	P	2450/2821	30–79	44/46	0.38	10
Park (2006)^19^	Korea	Asian	CRC	P	190/318	55/55	48/NR	0.42	7
Flugge (2007)^20^	Germany	Caucasian	CRC	H	256/256	61.9/62.2	52/51	0.41	7
Grunhage (2008)^21^	Germany	Caucasian	CRC	H	192/220	65/63	41/53	0.32	7
Naderi (2008)^35^	Iran	NR	UC, CD	P	230/150	35/35	34/34	0.26	7
Ochs-Balcom (2008)^22^	USA	Caucasian^(a)^	CRC	P	250/246	62.8/58.5	52/67	0.39	9
Theodoratou (2008)^23^	Scotland	Caucasian	CRC	P	2940/3038	62.0/62.4	43/43	0.39	9
Jenab (2009)^12^	Europe	Caucasian	CRC	P	1248/1248	58/58	50/50	0.39	10
Hughes (2011)^36^	Ireland	Caucasian	UC, CD	P	660/693	41.2/40.2	56/55	0.35	7
Mahmoudi (2011)^24^	Iran	NR	CRC	H	452/452	53.8/44.3	45/52	0.25	7
Pei (2011)^13^	China	Asian^(a)^	UC	P	218/250	39.4/41.6	35/44	0.39	8
Bentley (2012)^25^	New Zealand	Caucasian	CRC	P	200/200	69.5	47/47	0.38	6
Yamaji (2012)^32^	Japan	Asian^(a)^	CA	P	684/641	40–79	33/35	0.34	9
Rasool (2013)^26^	India	NR	CRC	P	312/305	52.1/51.1	45/49	0.26	7
Laczmanska (2014)^27^	Poland	Caucasian^(a)^	CRC	P	164/182	65.7/NR	41/41	0.42	7
Sarkissyan (2014)^28^	USA	Hispanic, African, Caucasian, Asian	CRC	H	78/230	55.1/54.9	45/63	0.36	7
Takeshige (2015)^29^	Japan	Asian^(a)^	CRC	P	685/778	60.2/58.6	38/37	0.37	9
Xia (2015)^37^	China	Asian^(a)^	UC	P	382/489	42.1/41	40/45	0.43	7
Alkhayal (2016)^30^	Saudi Arabia	Saudi Arabian	CRC	P	100/100	57.5^b^	36/35	0.24	8
Beckett (2016)^33^	Australia	Caucasian^(a)^	CA	P	57/201	66.2/61.1	46/59	0.35	8
Xia (2016)^39^	China	Asian^(a)^	CD	NR	297/446	27.1/28.2	45/46	0.43	7
Cho (2017, current)	Korea	Asian	CRC	P	701/1402	56.4/56.0	32/32	0.43	8
Zheng (2017)^38^	China	Asian^(a)^	UC	P	404/612	42.1/40.8	55/54	0.43	7

^(a)^No information on race in the paper. The race was hypothesized based on the more frequent ethnicity in the study country; ^(b)^Median. Abbreviations: CA, colorectal adenoma; CD, Crohn’s disease; CRC, colorectal cancer; H, hospital-based; MAF, minor allele frequency (f allele); NR, not reported; P, population-based; UC, ulcerative colitis.

**Figure 1:**
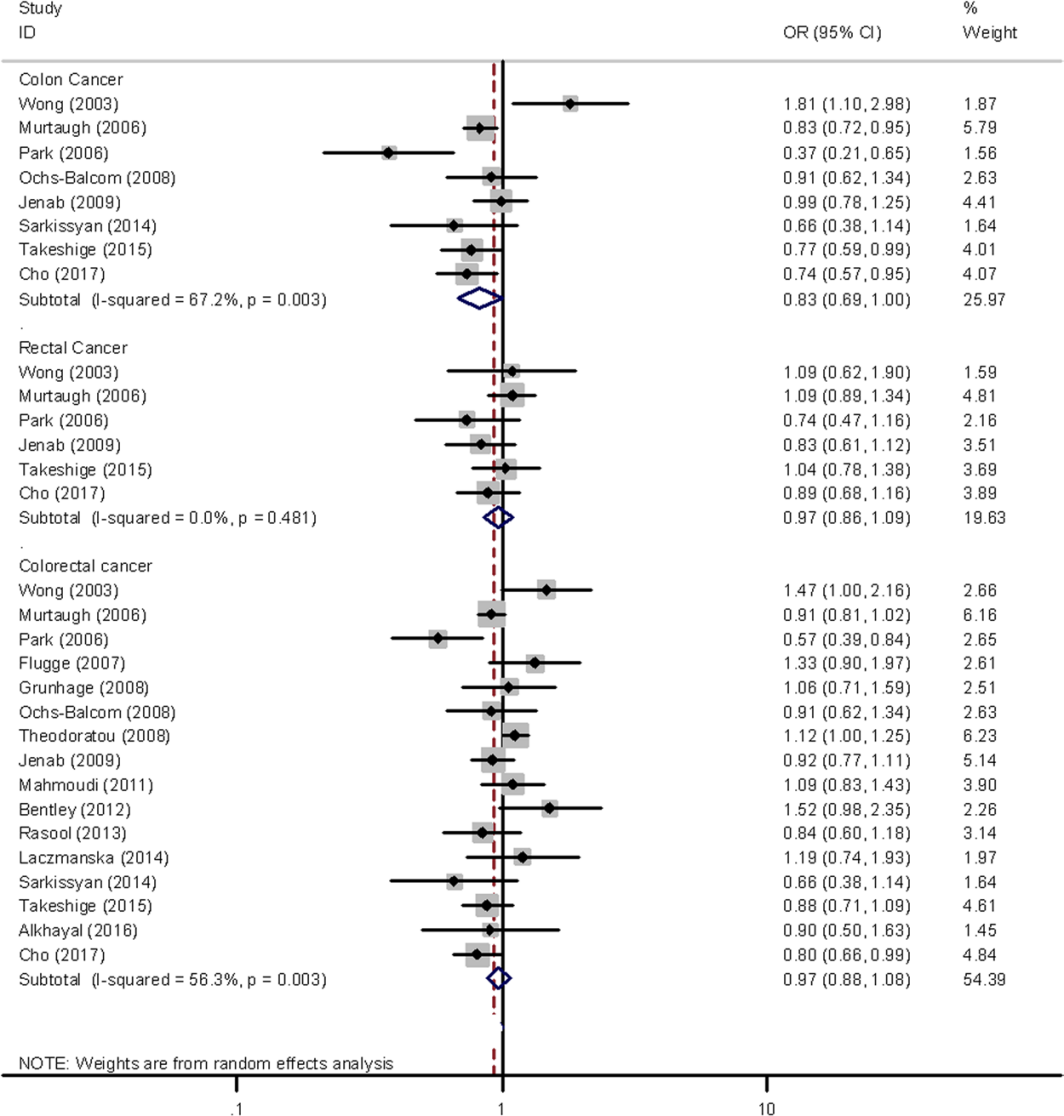
Forest plot showing the risk of colorectal cancer associated with *VDR Fok*I polymorphism (Ft vs. FF).

**Figure 2:**
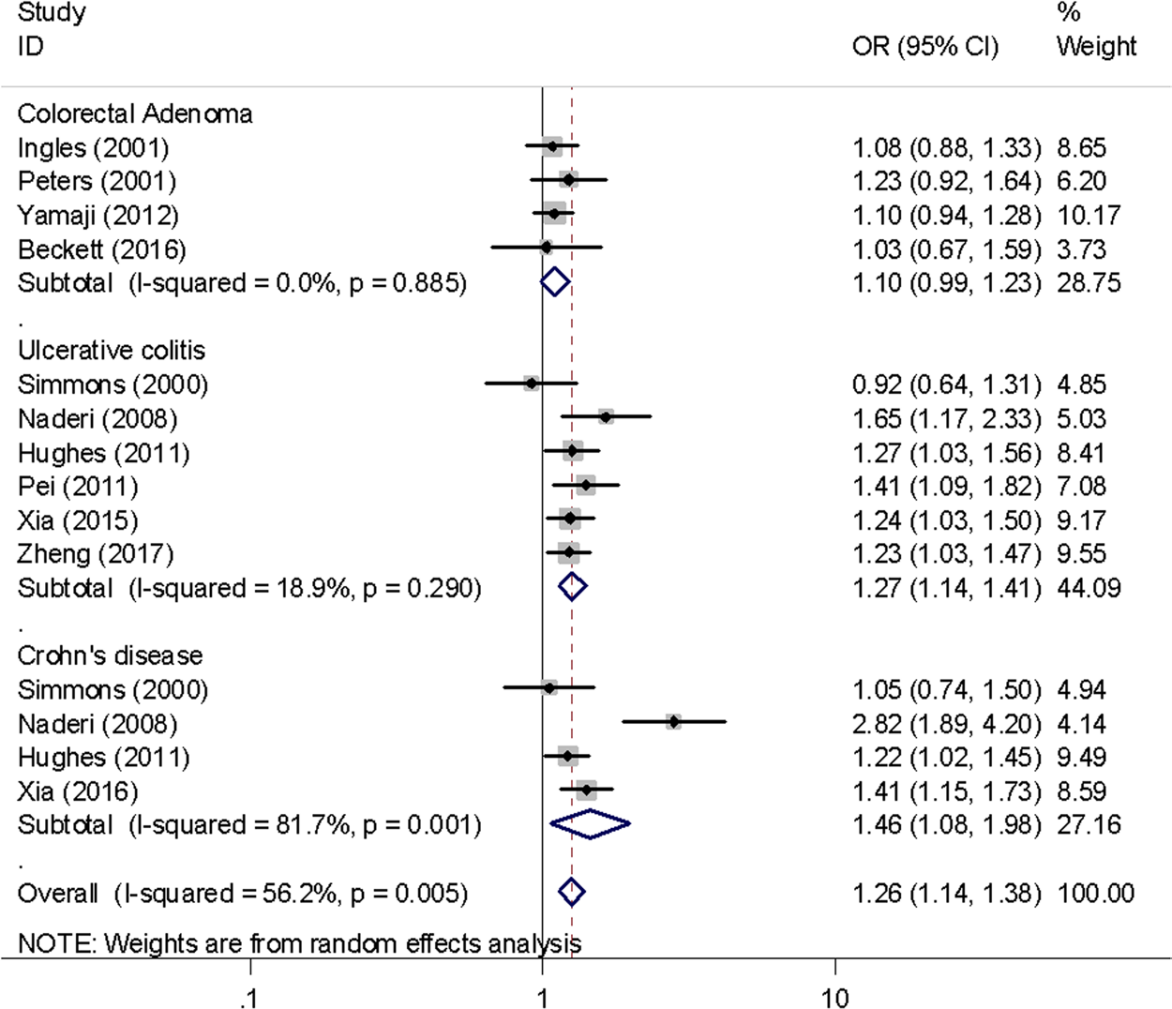
Forest plot showing the risk of colorectal adenoma and inflammatory bowel disease associated with *VDR Fok*I polymorphism (f allele vs. F allele).

The meta-analysis was conducted to investigate the association between *VDR Fok*I polymorphism and colorectal disease (Supplementary Table 2, Figs 1 and 2). *VDR Fok*I polymorphism was not significantly associated with colorectal cancer risk. However, a borderline significant association with a lower risk of colon cancer was observed for heterozygous carriers compared to homozygous carriers of the F allele (OR = 0.83, 95% CI = 0.69–1.00, *P* = 0.049 for Ff vs. FF). This polymorphism was not associated with rectal cancer. However, carriers of the f allele showed an increased risk of inflammatory bowel disease (OR = 1.38, 95% CI = 1.06–1.78, *P* = 0.015 for ff vs. FF; OR = 1.32, 95% CI = 1.17–1.50, *P* < 0.001 for f allele vs. F allele), both Crohn’s disease (OR = 1.46, 95% CI = 1.08–1.98, *P* = 0.015 for f allele vs F allele) and ulcerative colitis (OR = 1.27, 95% CI = 1.14–1.41, *P* < 0.001 for f allele vs. F allele). No significant association was observed for colorectal adenoma (OR = 1.11, 95% CI = 0.99–1.23, *P* = 0.077 for f allele vs. F allele), although the direction of the association was similar to that for inflammatory bowel disease.

Significant heterogeneity was observed in the associations with colorectal cancer, colon cancer, inflammatory bowel disease, and Crohn’s disease, as presented by Q-statistics (Supplementary Table 2) and I^2^ statistics (data not shown). Therefore, we conducted subgroup analyses and sensitivity analyses. When stratified by geographic location, the association between the f allele and colon cancer was stronger in studies conducted in non-Asian countries with low heterogeneity (OR = 0.86, 95% CI = 0.77–0.96, *P* = 0.010 for Ff vs. FF; *P* for heterogeneity = 0.452). Generally, heterogeneity was lower in non-Asian studies (Supplementary Table 3). In sensitivity analyses, excluding the study of Naderi *et al*.^35^ resulted in decreased heterogeneity in studies of Crohn’s disease with a significant positive association (OR = 1.26, 95% CI = 1.10–1.45, *P* = 0.001 for f allele vs. F allele; *P* for heterogeneity = 0.312). Additionally, excluding the study of Wong *et al*.^18^ resulted in a stronger inverse association and decreased the heterogeneity in colon cancer studies (OR = 0.79, 95% CI = 0.67–0.92, *P* = 0.002 for Ff vs. FF; *P* for heterogeneity = 0.069). However, other sensitivity analyses did not show any meaningful changes in significance or heterogeneity (Supplementary Table S4).

Visual inspection of the funnel plot revealed no publication bias (data not shown). Additionally, the results of Egger’s test indicated no significant publication bias (Supplementary Table 2).

## Discussion

The present study including both a Korean case-control study and a meta-analysis of 27 studies suggested that *VDR Fok*I polymorphism may have different associations with colorectal disease according to the type and severity of the disease.

To investigate the association between *VDR Fok*I and colorectal cancer, we conducted both a case-control study and a meta-analysis of 16 previous studies. In the current case-control study, we found no association of the *VDR Fok*I f allele with colorectal cancer. However, when cases were stratified by anatomic location and cancer stage, slightly inverse associations were observed with colon cancer and advanced-stage colorectal cancer. The inverse association of *Fok*I polymorphism with colon or advanced-stage colorectal cancer was much stronger in a previous Korean study^19^ and was reported in other studies conducted in the USA^11,28^. Park *et al*.^19^ found that the f allele was less frequent among patients with advanced cancer stage and lymph node metastasis. However, Wong *et al*.^18^ reported the opposite association to other previous studies. In their study, the f allele was associated with an increased risk of colorectal cancer, but other studies reported positive or non-significant associations^12,20–27,29,30^. Therefore, to clarify these inconsistencies, we conducted a meta-analysis of 16 studies including the present case-control study. No significant association was observed, except for a borderline significant association with a lower risk of colon cancer among heterozygous carriers compared to homozygous carriers of the F allele. This association was stronger in studies conducted in non-Asian countries. The possible association with colon cancer but not rectal cancer was consistent with the findings of the present case-control study. The possible inverse association with colon cancer but not with rectal cancer, may derive from differences in the VDR distribution in these tissues^40^.

The associations of *Fok*I polymorphism with precancerous diseases such as inflammatory bowel disease and colorectal adenoma was examined in this meta-analysis. An increased risk of inflammatory bowel disease was observed among *Fok*I f allele carriers compared to F allele carriers in the present meta-analysis. Several studies have reported significant positive associations of *Fok*I polymorphism with Crohn’s disease^35,36,39^ and ulcerative colitis^13,35–38^. In a Chinese case-control study^38^, the *Fok*I f allele was more frequently observed in patients with severe ulcerative colitis than in those with mild or moderate ulcerative colitis. Additionally, several previous studies have examined the *VDR Fok*I variant in relation to colorectal adenoma^14,31–33^, a precursor lesion for colorectal cancer. The present meta-analysis showed that *VDR Fok*I polymorphism had no association with the risk of colorectal adenoma, although the direction of the association was similar to that for inflammatory bowel disease.

Inflammatory bowel disease is a chronic and non-specific inflammatory disease of the gastrointestinal tract resulting from inappropriate function of the mucosal immune system^41^. Vitamin D and VDR are suggested to protect the intestine from damage by maintaining epithelial barrier function and decreasing mucosal inflammation. The shorter allele (F) of the *Fok*I polymorphism was reported to be more efficient at transactivating vitamin D-target genes and is thus expected to transmit stronger anti-proliferative and pro-differentiation signals^18^. Therefore, the *Fok*I f allele may influence the immune-regulatory function of the VDR^38,42^. Based on this relationship, the less active ff genotype would be expected to mimic the cellular consequences of lower vitamin D levels and may therefore increase the risk of disease. Several studies^6,43^ reported an inverse correlation between the circulating 25(OH)D concentration and the risk of inflammatory bowel disease. Based on this information, the f allele may cause vitamin D deficiency, thus increasing the risk of inflammatory bowel disease. In the present study, the *Fok*I f allele was positively associated with the risk of inflammatory bowel disease, which is consistent with the proposed mechanism.

However, the mechanism by which the *Fok*I polymorphism influences susceptibility to colorectal cancer may be more complex and is poorly understood. The *Fok*I f allele is suggested to be inversely associated with colon cancer and advanced colorectal cancer in the present case-control study and with colon cancer in the present meta-analysis, which is different from the findings for inflammatory bowel disease. Previous evidence suggests that the role of the *Fok*I polymorphism may differ by disease severity. First, VDR expression is reported to differ according to disease status^44,45^. A case-control study examining Puerto Rican patients reported a relationship between serum vitamin D levels, colonic VDR expression, and histologic disease activity^44^. Although colonic VDR expression in normal mucosa is positively correlated with serum vitamin D levels, VDR expression was significantly depressed in sporadic dysplasia and colorectal cancer tissues compared to that in normal and colitis-associated colorectal cancer tissues^44,45^. VDR expression in diseased colon tissues was associated with higher histologic inflammation scores, which reflect disease activity. Notably, the disease process interferes with the vitamin D-VDR system in diseased intestinal mucosa^44^. VDR expression has also been suggested to be up-regulated in early-stage cancer but not in advanced cancer^19^. Second, the role of vitamin D in carcinogenesis is influenced by tumor-host interactions. Although most vitamin D hydroxylation occurs in the liver and kidney, other tissues including intestinal epithelial cells and immune cells express vitamin D hydroxylase enzymes, suggesting that these cells can regulate local levels of the active hormone^46^. Because colorectal cancer with little lymphocytic infiltrate may not have sufficient bioactive vitamin D, the inverse association between vitamin D and colorectal cancer may be stronger for cancers with high-level immune responses than that for cancer with low-level immune responses. A nested case-control study conducted in the USA^47^ reported that high plasma 25(OH)D levels are associated with a lower risk of colorectal cancer with an intense immune response. Therefore, host immunity and disease status may help predict the benefit of vitamin D and the role of the VDR in colorectal carcinogenesis. Future studies are required to elucidate the complex network that exists between VDR expression, 25(OH)D levels, *Fok*I polymorphism, and patient health status.

The present case-control study has some limitations that should be considered when interpreting the findings. Because this study applied a case-control design, both recall and selection biases may be present. In the current case-control study, the controls were selected among those who voluntarily participated in a health check-up program; therefore, they may have been more health conscious than the general population. Additionally, subjects with inflammatory bowel disease and colorectal adenoma may have been included among the controls because the controls did not undergo endoscopy examination.

The meta-analysis also has several important limitations. First, the meta-analysis revealed significant heterogeneity for colorectal cancer, colon cancer, inflammatory bowel disease, and Crohn’s disease but not for rectal cancer, ulcerative colitis, and colorectal adenoma. High heterogeneity across studies may undermine the strength of the findings. The different results between studies may have resulted from the clinical heterogeneity of colorectal disease, different demographic and genetic characteristics of the study populations, different sources of controls, different environment and lifestyle factors including vitamin D status, and the reliability of the genotyping data. To identify the cause of heterogeneity, we conducted subgroup analyses and sensitivity analyses; however, the exact sources of heterogeneity remain unclear. Second, the association with only one polymorphism was examined; therefore, other *VDR* polymorphisms should be considered in the future. Third, some inevitable publication bias may be present because only published studies were used, although no publication bias was observed. Fourth, this meta-analysis was based on unadjusted OR estimates because not all of the studies reported adjusted ORs. In addition, in studies in which adjusted ORs were presented, the ORs were not adjusted by the same potential confounders. Finally, the small number of studies included in the meta-analysis limits the ability to draw a significant conclusion, especially in subgroup analyses

In conclusion, we found that the role of the *Fok*I polymorphism differs by the type and severity of colorectal disease. The *Fok*I f allele was associated with an increased risk of inflammatory bowel disease and may be associated with a decreased risk of colon cancer. Based on this relationship, any impairment of the 1,25(OH)_2_D_3_/VDR system (e.g., vitamin D deficiency, *VDR* polymorphism, or impaired intestinal function) can be assumed to alter the development or progression of colorectal disease. Therefore, additional well-designed studies are needed to confirm the exact role of the *VDR Fok*I polymorphism in colorectal diseases.

## Methods

### Case-Control Study

#### Study Subjects

This study has been described in detail elsewhere^48^. The eligible cases were newly diagnosed colorectal cancer patients at the Center for Colorectal Cancer of the National Cancer Center, Korea, between August 2010 and August 2013 who underwent surgery for colorectal cancer. Among the 1,070 patients who agreed to participate in the study, 369 were excluded because of incomplete semi-quantitative food frequency questionnaires (SQFFQs) or other questionnaires or implausible energy intakes (<500 or >4,000 kcal/day). Therefore, 923 patients were included in the analysis. The control subjects were recruited between October 2007 and December 2014 among individuals visiting the Center for Cancer Prevention and Detection at the same hospital for a health check-up program provided by the National Health Insurance Cooperation, which covers the entire Korean population. Of the 14,201 subjects who agreed to participate in the study, 5,164 were excluded because of incomplete SQFFQs or other questionnaires and implausible energy intakes (<500 or >4,000 kcal/day). The data of the remaining subjects were linked with the Korea Central Cancer Registry and National Cancer Center medical chart to confirm that these subjects had not been diagnosed with colorectal cancer. Of the remaining 9,037 individuals, two controls per case were randomly selected and frequency-matched by sex and 5-year age group. Therefore, 923 cases and 1,846 controls were originally selected to investigate the association between environmental factors and colorectal cancer risk. However, among 923 cases, 222 were missing blood samples. Therefore, 1:2 matching was conducted again, and 701 cases and 1,402 controls were selected for genotyping. After genotyping, 6 cases and 5 controls were excluded because of genotyping failure. Ultimately, 695 colorectal cancer patients and 1,397 healthy controls were selected for the final analysis (Supplementary Fig. S2).

All participants provided written informed consent, and the study protocol was approved by the Institutional Review Board of the National Cancer Center (IRB No. NCCNCS-10-350 and NCC2015-0202). All procedures used in the present study were performed in accordance with the guidelines and regulations of the IRB of the National Cancer Center.

#### Data Collection and Genotyping

A trained interviewer conducted face-to-face interviews to collect information on lifestyle factors and dietary habits prior to cancer diagnosis. Information regarding the participants’ demographic and lifestyle risk factors (e.g., smoking, alcohol drinking, and regular exercise) was collected using a structured questionnaire.

The *VDR Fok*I polymorphism (rs2228570) was genotyped as described below. Genomic DNA was extracted using the MagAttract DNA Blood M48 kit (Qiagen, Hilden, Germany) and BioRobot M48 automatic extraction equipment (Qiagen) according to the manufacturer’s instructions. Genotyping was performed using a MassARRAY iPLEX Gold Assay (Agena Bioscience, San Diego, CA). To control genotyping quality, duplicate samples for 3% of the subjects were included in our initial genotyping analysis; the rate of discordance was <1%. Genotyping was successfully performed for 695 cases and 1,397 controls.

#### Statistical analysis

Differences in the demographic and lifestyle factors between the cases and controls were analyzed using the χ^2^ test for categorical variables and Student’s t-test for continuous variables. The χ^2^ test was used to test for Hardy-Weinberg Equilibrium (HWE) of the *VDR Fok*I polymorphism in the control group. The association between *VDR Fok*I polymorphism and colorectal cancer risk was analyzed using unconditional logistic regression models. A multivariable model was adjusted for age (continuous), sex (men/women), body mass index (BMI) (<25, ≥25 kg/m^2^), education (middle school or less, high school, college or more), family history of colorectal cancer (yes/no), regular exercise (yes/no), and total caloric intake (continuous). To determine which variables to enter into the multivariable model, we performed backward selection using colorectal risk factors, which were selected based on both our data and prior information. A multinomial logistic regression model was used for analyses stratified by anatomic location (colon and rectal cancer) and cancer stage. American Joint Committee on Cancer (AJCC) stages III and IV were considered advanced cancer.

All statistical analyses were performed using SAS 9.2 (SAS Institute Inc., Cary, NC). A two-sided *P*-value of less than 0.05 indicated statistical significance.

### Meta-analysis of Published Studies

#### Literature Search

This meta-analysis was performed according to the PRISMA guidelines. To identify all articles exploring the association between *VDR Fok*I polymorphism and the risk of colorectal cancer, colorectal adenoma, or inflammatory bowel disease, we conducted a literature search using PubMed and EMBASE through March 2017. We used the following search terms: (1) “*VDR* polymorphism”, rs2228570, rs10735810, or *Fok*I; (2) “colorectal cancer”, “colon cancer”, “rectal cancer”, “colorectal adenoma”, “inflammatory bowel disease”, “ulcerative colitis” or “Crohn’s disease”. Search terms included both MeSH terms and text words. To identify additional studies, we also screened the references of the retrieved publications and review papers. This search was limited to human studies and publications written in English. We did not consider abstracts or unpublished reports.

#### Inclusion Criteria

The studies included in this meta-analysis were required to meet the following criteria: (1) investigated the association between *VDR Fok*I polymorphism and the abovementioned diseases; (2) provided genotype frequencies for cases and controls such that ORs with 95% CIs and HWE could be calculated; and (3) featured a control population genotype distribution that did not deviate from HWE (*P* > 0.01). If duplicated data were reported in multiple publications, the most complete publication was selected.

#### Data Extraction and Quality Assessment

The following information was extracted from each article: author name, year of publication, country of study, ethnicity, source of the controls, mean age, proportion of women, vitamin D status, and genotype frequencies for cases and controls.

Predefined criteria (Supplementary Table S5) based on the scale of Thakkinstian *et al*.^49^ were used to assess the methodological quality of eligible studies (Supplementary Table S6). The revised criteria include the representativeness of cases and controls, assessment of colorectal disease, genotyping examination, HWE deviation in the control population, and association assessment. Scores ranged from 0 (lowest) to 11 (highest). Articles with scores of less than 6 were considered to be low-quality studies, whereas those with scores equal to or higher than 6 were considered high-quality reports. Data extraction and quality assessment were performed by two investigators independently. Disagreements were resolved by consensus.

#### Statistical analysis

The strengths of associations between *VDR Fok*I polymorphism and selected disease risk (colorectal cancer, colorectal adenoma, and inflammatory bowel disease) were measured using unadjusted ORs with corresponding 95% CIs, which were calculated based on genotype frequencies. Forest plots were used to illustrate the results of the included studies. Four different ORs were calculated using the following models: (1) homozygote comparison, (2) heterozygote comparison, (3) dominant genetic model, and (4) allele comparison. Before analysis, the genotype frequencies of the polymorphisms were assessed for HWE using the chi-squared test. A *P*-value less than 0.01 indicated a significant deviation from HWE. The summary OR estimated for each study was calculated using a random effects model. Crohn’s disease and ulcerative colitis were assessed together or separately. Heterogeneity among the included articles was estimated using the Q-statistic and *I*^2^ statistic. Potential sources of heterogeneity were sought via subgroup analyses by geographic location and anatomic location. We also performed sensitivity analyses by excluding one study each. Publication bias was assessed by visual inspection of funnel plots and formal statistical assessment of funnel plot asymmetry was performed with Egger’s regression test. Publication bias was considered present if the *P*-value of the intercept was less than 0.05.

All statistical analyses were performed using STATA software (version 14; Stata Corporation, College Station, Texas). Two-sided *P-*values less than 0.05 indicated statistical significance.

## Electronic supplementary material

Supplementary Material

## Data Availability

The dataset generated and/or analyzed during the present study are available from the corresponding author on reasonable request.
