# Supplementary material for: Vitamin D receptor FokI polymorphism and the risks of colorectal cancer, inflammatory bowel disease, and colorectal adenoma
Source: Sci Rep. 2018 Aug 27;8:12899. doi: 10.1038/s41598-018-31244-5 (PMC6110797; doi:10.1038/s41598-018-31244-5)

# Vitamin D receptor *Fok*I polymorphism and the risks of colorectal cancer, inflammatory bowel disease, and colorectal adenoma

Young Ae Cho[1], Jeonghee Lee[1], Jae Hwan Oh[2], Hee Jin Chang[2], Dae Kyung Sohn[2], Aesun Shin[1,3] & Jeongseon Kim[1*]

[1]Department of Cancer Biomedical Science, Graduate School of Cancer Science and Policy, National Cancer Center, Goyang, South Korea. [2]Center for Colorectal Cancer, National Cancer Center Hospital, National Cancer Center, South Korea. [3]Department of Preventive Medicine, Seoul National University College of Medicine, Seoul, South Korea

***Corresponding author:** Jeongseon Kim (e-mail: jskim@ncc.re.kr)

**Supplementary Table S1. General characteristics of the study subjects.** [(a)]First-degree relative. Abbreviations: BMI, body mass index.

| | **Controls (n = 1397)** | **Cases (n = 695)** | ***P*-value** |
|---|---|---|---|
| Age (years), mean ± SD | 56.0 ± 9.1 | 56.5 ± 9.5 | 0.236 |
| Female, n (%) | 442 (31.6) | 221 (31.8) | 0.941 |
| Family history of colorectal cancer[(a)] (yes), n (%) | 77 (5.5) | 66 (9.9) | <0.001 |
| BMI ($kg/m^2$), n (%) | | | |
| <25 | 931 (66.6) | 475 (68.4) | 0.435 |
| ≥25 | 466 (33.4) | 220 (31.7) | |
| Education level, n (%) | | | |
| Middle school or less | 196 (14.1) | 253 (36.4) | <0.001 |
| High school | 454 (32.8) | 265 (38.1) | |
| College or more | 736 (53.1) | 177 (25.5) | |
| Smoking status, n (%) | | | |
| Nonsmoker | 613 (43.9) | 313 (45.0) | 0.616 |
| Ever smoker | 7847 (56.1) | 382 (55.0) | |
| Alcohol consumption, n (%) | | | |
| Nondrinker | 419 (30.0) | 211 (30.4) | 0.863 |
| Ever drinker | 978 (70.0) | 484 (69.6) | |
| Regular exercise (yes), n (%) | 828 (59.6) | 562 (40.4) | <0.001 |
| Total caloric intake (kcal/day), mean ± SD | 1698.9 ± 560.8 | 2020.1 ± 531.1 | <0.001 |

**Supplementary Table S2. Meta-analysis of the risk of colorectal disease associated with *VDR Fok*I polymorphism**. Abbreviations: CI, confidence interval; OR, odds ratio; VDR, vitamin D receptor.

| ***VDR Fok*I** | **No. Studies** | **Test for association** | | ***P*-value for heterogeneity** | ***P*-value for Egger's test** |
|---|---|---|---|---|---|
| | | **OR (95% CI)** | ***P*-value** | | |
| **Colorectal cancer** | 16 | | | | |
| ff *vs*. FF | | 0.99 (0.85–1.15) | 0.853 | 0.003 | 0.680 |
| Ff *vs*. FF | | 0.97 (0.88–1.08) | 0.601 | 0.003 | 0.236 |
| Ff+ff *vs*. FF | | 0.97 (0.88–1.08) | 0.619 | <0.001 | 0.921 |
| F allele *vs*. F allele | | 0.98 (0.91–1.06) | 0.673 | <0.001 | 0.864 |
| **Colon cancer** | 8 | | | | |
| ff *vs*. FF | | 0.88 (0.65–1.21) | 0.442 | <0.001 | 0.882 |
| Ff *vs*. FF | | 0.83 (0.69–1.00) | 0.049 | 0.003 | 0.864 |
| Ff+ff *vs*. FF | | 0.83 (0.67–1.02) | 0.080 | <0.001 | 0.970 |
| f allele *vs*. F allele | | 0.90 (0.76–1.05) | 0.174 | <0.001 | 0.912 |
| **Rectal cancer** | 6 | | | | |
| ff *vs*. FF | | 0.92 (0.71–1.18) | 0.489 | 0.082 | 0.292 |
| Ff *vs*. FF | | 0.97 (0.86–1.09) | 0.567 | 0.048 | 0.367 |
| Ff+ff *vs*. FF | | 0.95 (0.82–1.09) | 0.436 | 0.245 | 0.402 |
| f allele *vs*. F allele | | 0.96 (0.85–1.08) | 0.470 | 0.080 | 0.381 |
| **Inflammatory bowel disease** | 10 | | | | |
| ff *vs*. FF | | 1.38 (1.06–1.78) | 0.015 | 0.009 | 0.093 |
| Ff *vs*. FF | | 1.08 (0.91–1.29) | 0.386 | 0.029 | 0.884 |
| Ff+ff *vs*. FF | | 1.16 (0.96–1.40) | 0.137 | 0.005 | 0.695 |
| f allele *vs*. F allele | | 1.32 (1.17–1.50) | <0.001 | 0.005 | 0.333 |
| **Crohn's disease** | 4 | | | | |
| ff *vs*. FF | | 1.73 (0.93–3.21) | 0.084 | 0.002 | 0.339 |
| Ff *vs*. FF | | 1.13 (0.78–1.65) | 0.518 | 0.024 | 0.884 |
| Ff+ff *vs*. FF | | 1.27 (0.83–1.95) | 0.272 | 0.003 | 0.674 |
| f allele *vs*. F allele | | 1.46 (1.08–1.98) | 0.015 | 0.001 | 0.475 |
| **Ulcerative colitis** | 6 | | | | |
| ff *vs*. FF | | 1.22 (0.97–1.52) | 0.088 | 0.303 | 0.352 |
| Ff *vs*. FF | | 1.06 (0.86–1.31) | 0.584 | 0.110 | 0.607 |
| Ff+ff *vs*. FF | | 1.07 (0.90–1.36) | 0.329 | 0.096 | 0.815 |
| f allele *vs*. F allele | | 1.27 (1.14–1.41) | <0.001 | 0.290 | 0.909 |
| **Colorectal adenoma** | 4 | | | | |
| ff *vs*. FF | | 0.95 (0.74–1.21) | 0.658 | 0.748 | 0.933 |
| Ff *vs*. FF | | 1.05 (0.89–1.24) | 0.536 | 0.836 | 0.734 |
| Ff+ff *vs*. FF | | 1.03 (0.88–1.20) | 0.714 | 0.772 | 0.580 |
| f allele *vs*. F allele | | 1.11 (0.99–1.23) | 0.077 | 0.885 | 0.857 |

**Supplementary Table S3. Meta-analysis of the risk of colorectal disease associated with *VDR Fok*I polymorphism, stratified by geographic location.** Only meta-analyses of colorectal diseases showing significant heterogeneity are presented. Abbreviations: CI, confidence interval; OR, odds ratio; VDR, vitamin D receptor.

| ***VDR Fok*I** | **No. Studies** | **Test for association** | | ***P*-value for heterogeneity** | **No. Studies** | **Test for association** | | ***P*-value for heterogeneity** |
|---|---|---|---|---|---|---|---|---|
| | | **OR (95% CI)** | ***P*-value** | | | **OR (95% CI)** | ***P*-value** | |
| **Geographic location** | **Asia** | | | | **Non-Asia** | | | |
| **Colorectal cancer** | 7 | | | | 9 | | | |
| ff *vs*. FF | | 0.94 (0.67–1.31) | 0.702 | 0.002 | | 1.00 (0.86–1.18) | 0.975 | 0.086 |
| Ff *vs*. FF | | 0.90 (0.75–1.08) | 0.265 | 0.023 | | 1.03 (0.91–1.16) | 0.674 | 0.047 |
| Ff+ff *vs*. FF | | 0.91 (0.74–1.11) | 0.357 | 0.003 | | 1.02 (0.91–1.15) | 0.733 | 0.026 |
| F allele *vs*. F allele | | 0.95 (0.81–1.11) | 0.481 | 0.001 | | 1.01 (0.93–1.09) | 0.867 | 0.033 |
| **Colon cancer** | 4 | | | | 4 | | | |
| ff *vs*. FF | | 0.85 (0.49–1.47) | 0.567 | 0.002 | | 0.90 (0.57–1.43) | 0.654 | 0.002 |
| Ff *vs*. FF | | 0.79 (0.51–1.23) | 0.293 | 0.001 | | 0.86 (0.77–0.96) | 0.010 | 0.452 |
| Ff+ff *vs*. FF | | 0.79 (0.50–1.25) | 0.322 | <0.001 | | 0.87 (0.71–1.07) | 0.193 | 0.069 |
| f allele *vs*. F allele | | 0.86 (0.63–1.16) | 0.325 | <0.001 | | 0.92 (0.75–1.14) | 0.445 | 0.002 |
| **Inflammatory bowel disease** | 5 | | | | 5 | | | |
| ff *vs*. FF | | 1.74 (1.06–2.85) | 0.028 | 0.001 | | 1.18 (0.94–1.48) | 0.160 | 0.725 |
| Ff *vs*. FF | | 1.17 (0.92–1.50) | 0.210 | 0.094 | | 0.98 (0.73–1.31) | 0.884 | 0.033 |
| Ff+ff *vs*. FF | | 1.33 (0.97–1.83) | 0.076 | 0.006 | | 1.03 (0.81–1.32) | 0.804 | 0.077 |
| f allele *vs*. F allele | | 1.50 (1.21–1.86) | <0.001 | 0.003 | | 1.21 (1.08–1.36) | 0.001 | 0.349 |
| **Crohn's disease** | 2 | | | | 2 | | | |
| ff *vs*. FF | | 3.00 (0.62–14.49) | 0.171 | 0.001 | | 1.13 (0.81–1.57) | 0.483 | 0.941 |
| Ff *vs*. FF | | 1.51 (0.70–3.28) | 0.296 | 0.031 | | 0.90 (0.51–1.58) | 0.712 | 0.062 |
| Ff+ff *vs*. FF | | 1.84 (0.70–4.80) | 0.215 | 0.004 | | 0.96 (0.63–1.49) | 0.870 | 0.125 |
| f allele *vs*. F allele | | 1.95 (0.99–3.83) | 0.053 | 0.003 | | 1.18 (1.01–1.38) | 0.053 | 0.474 |

**Supplementary Table S4. Sensitivity analyses deleting individual studies to determine the influence of the individual study on the pooled ORs.**. Only meta-analyses of colorectal diseases showing significant heterogeneity are presented. [(a)] *P*-value for association. [(b)] *P*-value for heterogeneity. Abbreviations: CD, Crohn's disease; CI, confidence interval; OR, odds ratio; UC, ulcerative colitis; VDR, vitamin D receptor.

| Study omitted | ***VDR FokI*** | | | | | | | | | | | |
|---|---|---|---|---|---|---|---|---|---|---|---|---|
| | **Ff vs. FF** | | | **Ff vs. FF** | | | **Ff+ff vs. FF** | | | **F allele vs. F allele** | | |
| | **Test for association** | | $P^{(b)}$ | **Test for association** | | $P^{(b)}$ | **Test for association** | | $P^{(b)}$ | **Test for association** | | $P^{(b)}$ |
| | **OR (95% CI)** | $P^{(a)}$ | | **OR (95% CI)** | $P^{(a)}$ | | **OR (95% CI)** | $P^{(a)}$ | | **OR (95% CI)** | $P^{(a)}$ | |
| **Colorectal cancer (n=15)** | | | | | | | | | | | | |
| Wong (2003) | 0.94 (0.82–1.09) | 0.431 | 0.023 | 0.95 (0.86–1.06) | 0.356 | 0.008 | 0.95 (0.86–1.05) | 0.323 | 0.002 | 0.97 (0.90–1.04) | 0.336 | 0.003 |
| Murtaugh (2006) | 1.01 (0.86–1.20) | 0.878 | 0.009 | 0.98 (0.87–1.11) | 0.761 | 0.004 | 0.99 (0.87–1.11) | 0.805 | 0.001 | 0.99 (0.92–1.08) | 0.889 | 0.001 |
| Park (2006) | 1.02 (0.89–1.16) | 0.779 | 0.049 | 1.00 (0.90–1.10) | 0.922 | 0.020 | 1.00 (0.91–1.10) | 0.995 | 0.011 | 1.00 (0.94–1.07) | 0.895 | 0.024 |
| Flugge (2007) | 0.97 (0.83–1.14) | 0.694 | 0.003 | 0.96 (0.86–1.06) | 0.421 | 0.004 | 0.96 (0.86–1.07) | 0.431 | 0.001 | 0.97 (0.90–1.05) | 0.507 | 0.001 |
| Grunhage (2008) | 0.98 (0.84–1.15) | 0.825 | 0.002 | 0.97 (0.87–1.08) | 0.568 | 0.002 | 0.97 (0.87–1.08) | 0.581 | <0.001 | 0.98 (0.91–1.06) | 0.633 | <0.001 |
| Ochs-Balcom (2008) | 0.96 (0.83–1.13) | 0.647 | 0.004 | 0.98 (0.88–1.09) | 0.664 | 0.002 | 0.97 (0.87–1.09) | 0.605 | <0.001 | 0.98 (0.90–1.06) | 0.539 | <0.001 |
| Theodoratou (2008) | 0.99 (0.82–1.18) | 0.871 | 0.002 | 0.95 (0.86–1.06) | 0.378 | 0.022 | 0.96 (0.86–1.08) | 0.489 | 0.002 | 0.98 (0.90–1.07) | 0.634 | 0.001 |
| Jenab (2009) | 0.98 (0.83–1.16) | 0.812 | 0.002 | 0.98 (0.87–1.10) | 0.711 | 0.002 | 0.98 (0.87–1.10) | 0.689 | <0.001 | 0.98 (0.90–1.07) | 0.673 | <0.001 |
| Mahmoudi (2011) | 0.99 (0.85–1.17) | 0.926 | 0.002 | 0.96 (0.86–1.08) | 0.518 | 0.002 | 0.97 (0.86–1.08) | 0.563 | <0.001 | 0.98 (0.91–1.06) | 0.650 | <0.001 |
| Bentley (2012) | 0.98 (0.84–1.15) | 0.826 | 0.002 | 0.96 (0.86–1.06) | 0.376 | 0.007 | 0.96 (0.86–1.07) | 0.428 | 0.001 | 0.98 (0.91–1.06) | 0.573 | <0.001 |
| Rasool (2013) | 0.97 (0.83–1.14) | 0.732 | 0.003 | 0.98 (0.88–1.09) | 0.734 | 0.002 | 0.98 (0.87–1.09) | 0.696 | <0.001 | 0.98 (0.91–1.06) | 0.670 | <0.001 |
| Laczmanska (2014) | 0.97 (0.83–1.14) | 0.734 | 0.003 | 0.97 (0.87–1.07) | 0.522 | 0.002 | 0.97 (0.87–1.08) | 0.519 | <0.001 | 0.98 (0.90–1.06) | 0.561 | <0.001 |
| Sarkissyan (2014) | 1.01 (0.87–1.17) | 0.952 | 0.007 | 0.98 (0.89–1.09) | 0.757 | 0.004 | 0.99 (0.89–1.10) | 0.833 | 0.001 | 1.00 (0.93–1.07) | 0.917 | 0.002 |
| Takeshige (2015) | 1.00 (0.85–1.18) | 0.966 | 0.002 | 0.98 (0.88–1.10) | 0.755 | 0.003 | 0.98 (0.88–1.10) | 0.786 | <0.001 | 0.99 (0.92–1.08) | 0.848 | <0.001 |
| Alkhayal (2016) | 0.98 (0.84–1.15) | 0.809 | 0.002 | 0.98 (0.88–1.09) | 0.641 | 0.002 | 0.97 (0.87–1.09) | 0.638 | <0.001 | 0.98 (0.91–1.06) | 0.663 | <0.001 |
| Cho (2017) | 1.00 (0.84–1.18) | 0.966 | 0.002 | 0.99 (0.89–1.10) | 0.874 | 0.007 | 0.99 (0.88–1.11) | 0.844 | 0.001 | 0.99 (0.91–1.08) | 0.811 | <0.001 |
| **Colon cancer (n=7)** | | | | | | | | | | | | |
| Wong (2003) | 0.81 (0.60–1.07) | 0.140 | 0.003 | 0.79 (0.67–0.92) | 0.002 | 0.068 | 0.78 (0.65–0.93) | 0.006 | 0.006 | 0.85 (0.73–0.99) | 0.035 | <0.001 |
| Murtaugh (2006) | 0.92 (0.63–1.33) | 0.646 | <0.001 | 0.82 (0.64–1.06) | 0.132 | 0.002 | 0.83 (0.63–1.10) | 0.192 | <0.001 | 0.90 (0.74–1.10) | 0.297 | <0.001 |
| Park (2006) | 0.96 (0.71–1.31) | 0.100 | 0.039 | 0.87 (0.74–1.03) | 0.100 | 0.039 | 0.89 (0.74–1.07) | 0.223 | 0.004 | 0.95 (0.82–1.10) | 0.473 | <0.001 |
| Ochs-Balcom (2008) | 0.83 (0.60–1.15) | 0.259 | <0.001 | 0.82 (0.66–1.01) | 0.059 | 0.002 | 0.81 (0.64–1.01) | 0.065 | <0.001 | 0.87 (0.73–1.03) | 0.097 | <0.001 |
| Jenab (2009) | 0.83 (0.59–1.18) | 0.300 | <0.001 | 0.80 (0.64–0.99) | 0.044 | 0.004 | 0.80 (0.63–1.01) | 0.058 | <0.001 | 0.87 (0.73–1.03) | 0.110 | <0.001 |

| | | | | | | | | | | | | |
|---|---|---|---|---|---|---|---|---|---|---|---|---|
| Sarkissyan (2014) | 0.94 (0.69–1.29) | 0.719 | <0.001 | 0.84 (0.69–1.03) | 0.096 | 0.002 | 0.86 (0.69–1.07) | 0.173 | <0.001 | 0.93 (0.79–1.09) | 0.365 | <0.001 |
| Takeshige (2015) | 0.88 (0.61–1.28) | 0.511 | <0.001 | 0.84 (0.67–1.05) | 0.118 | 0.002 | 0.84 (0.65–1.07) | 0.157 | <0.001 | 0.90 (0.74–1.08) | 0.246 | <0.001 |
| Cho (2017) | 0.90 (0.62–1.31) | 0.585 | <0.001 | 0.84 (0.68–1.05) | 0.130 | 0.002 | 0.85 (0.66–1.08) | 0.177 | <0.001 | 0.90 (0.75–1.09) | 0.274 | <0.001 |
| **Inflammatory bowel disease (n=9)** | | | | | | | | | | | | |
| Simmons (2000)-UC | 1.43 (1.09–1.87) | 0.010 | 0.008 | 1.13 (0.96–1.33) | 0.140 | 0.104 | 1.21 (1.01–1.45) | 0.041 | 0.019 | 1.36 (1.20–1.54) | 0.012 | <0.001 |
| Naderi (2008)-UC | 1.31 (1.01–1.70) | 0.039 | 0.015 | 1.05 (0.87–1.27) | 0.579 | 0.028 | 1.12 (0.92–1.36) | 0.265 | 0.007 | 1.30 (1.14–1.48) | <0.001 | 0.006 |
| Hughes (2011)-UC | 1.42 (1.06–1.90) | 0.019 | 0.005 | 1.05 (0.87–1.27) | 0.627 | 0.035 | 1.14 (0.92–1.41) | 0.235 | 0.004 | 1.34 (1.16–1.54) | <0.001 | 0.003 |
| Pei (2011)-UC | 1.36 (1.02–1.80) | 0.034 | 0.007 | 1.08 (0.88–1.31) | 0.464 | 0.018 | 1.15 (0.93–1.42) | 0.200 | 0.003 | 1.32 (1.15–1.51) | <0.001 | 0.003 |
| Xia (2015)-UC | 1.43 (1.07–1.92) | 0.017 | 0.006 | 1.10 (0.90–1.34) | 0.371 | 0.021 | 1.18 (0.95–1.46) | 0.137 | 0.004 | 1.34 (1.16–1.55) | <0.001 | 0.003 |
| Zheng (2017)-UC | 1.44 (1.07–1.93) | 0.015 | 0.008 | 1.10 (0.90–1.34) | 0.373 | 0.021 | 1.18 (0.95–1.46) | 0.134 | 0.004 | 1.34 (1.16–1.55) | 0.003 | <0.001 |
| Simmons (2000)-CD | 1.41 (1.07–1.85) | 0.016 | 0.005 | 1.12 (0.95–1.33) | 0.185 | 0.063 | 1.20 (0.99–1.45) | 0.065 | 0.009 | 1.35 (1.18–1.54) | <0.001 | 0.005 |
| Naderi (2008)-CD | 1.21 (1.03–1.42) | 0.018 | 0.568 | 1.04 (0.89–1.22) | 0.615 | 0.129 | 1.09 (0.94–1.26) | 0.240 | 0.152 | 1.26 (1.17–1.37) | <0.001 | 0.387 |
| Hughes (2011)-CD | 1.43 (1.06–1.93) | 0.019 | 0.006 | 1.07 (0.87–1.32) | 0.514 | 0.018 | 1.16 (0.93–1.45) | 0.195 | 0.003 | 1.34 (1.16–1.55) | <0.001 | 0.004 |
| Xia (2016)-CD | 1.38 (1.03–1.86) | 0.031 | 0.005 | 1.08 (0.89–1.33) | 0.432 | 0.017 | 1.16 (0.93–1.43) | 0.185 | 0.003 | 1.32 (1.14–1.51) | <0.001 | 0.004 |
| **Crohn's disease (n=3)** | | | | | | | | | | | | |
| Simmons (2000) | 2.02 (0.92–4.37) | 0.082 | 0.001 | 1.29 (0.90–1.84) | 0.160 | 0.082 | 1.49 (0.93–2.39) | 0.100 | 0.007 | 1.61 (1.12–2.33) | 0.011 | 0.001 |
| Naderi (2008) | 1.23 (0.95–1.59) | 0.124 | 0.720 | 0.99 (0.75–1.32) | 0.961 | 0.174 | 1.07 (0.85–1.34) | 0.573 | 0.275 | 1.26 (1.10–1.45) | 0.001 | 0.312 |
| Hughes (2011) | 2.13 (0.80–5.61) | 0.129 | 0.002 | 1.14 (0.61–2.16) | 0.679 | 0.009 | 1.35 (0.66–2.77) | 0.414 | 0.001 | 1.59 (0.99–2.55) | 0.056 | 0.001 |
| Xia (2016) | 1.97 (0.69–5.59) | 0.204 | 0.001 | 1.17 (0.65–2.12) | 0.597 | 0.009 | 1.34 (0.68–2.34) | 0.402 | 0.001 | 1.50 (0.91–2.48) | 0.109 | <0.001 |

**Supplementary Table S5. Scale for quality assessment of the single nucleotide polymorphism association studies of colorectal cancer, inflammatory bowel disease, and colorectal adenoma.**

| | **Criteria** | **Score** |
|---|---|---|
| **A** | **Representativeness of the cases** | |
| | Consecutive/randomly selected from a case population with a clearly defined sampling frame | 2 |
| | Consecutive/randomly selected from a case population without a clearly defined sampling frame or with extensive inclusion/exclusion criteria | 1 |
| | No method of selection described | 0 |
| **B** | **Representativeness of the controls** | |
| | Controls were consecutive/randomly drawn from the same sampling frame (ward/community) as the cases | 2 |
| | Controls were consecutive/randomly drawn from a different sampling frame as the cases | 1 |
| | Not described | 0 |
| **C** | **Ascertainment of colorectal disease** | |
| | Clearly described objective criteria for diagnosis of colorectal disease | 2 |
| | Diagnosis of colorectal disease by patient self-report or patient history | 1 |
| | Not described | 0 |
| **D** | **Genotyping examination** | |
| | Genotyping performed under a “blinded” condition | 1 |
| | Unblinded or not mentioned | 0 |
| **E** | **Hardy-Weinberg equilibrium** | |
| | Hardy-Weinberg equilibrium in the control group | 2 |
| | Hardy-Weinberg disequilibrium in the control group | 1 |
| | No assessment of Hardy-Weinberg equilibrium | 0 |
| **F** | **Association assessment** | |
| | Association between genotypes and colorectal disease assessed with appropriate statistics and adjustment for confounders | 2 |
| | Associations between genotypes and colorectal disease assessed with appropriate statistics without adjustment for confounders | 1 |
| | Inappropriate statistics used | 0 |

**Supplementary Table S6. Quality assessment of included studies.** A: representativeness of cases; B: representative of controls; C: ascertainment of colorectal disease; D: genotyping examination; E: Hardy-Weinberg equilibrium; F: association assessment.

| **First author (year)[ref]** | **A** | **B** | **C** | **D** | **E** | **F** | **Total score** |
|---|---|---|---|---|---|---|---|
| Simmons (2000)[34] | 1 | 1 | 2 | 0 | 2 | 1 | 7 |
| Ingles (2001)[14] | 1 | 2 | 2 | 1 | 2 | 2 | 10 |
| Peters (2001)[31] | 1 | 2 | 2 | 0 | 2 | 2 | 9 |
| Wong (2003)[18] | 1 | 1 | 2 | 0 | 2 | 2 | 8 |
| Murtaugh (2006)[11] | 2 | 2 | 2 | 0 | 2 | 2 | 10 |
| Park (2006)[19] | 1 | 1 | 2 | 0 | 2 | 1 | 7 |
| Flugge (2007)[20] | 1 | 1 | 2 | 0 | 2 | 1 | 7 |
| Grunhage (2008)[21] | 1 | 1 | 2 | 0 | 2 | 1 | 7 |
| Naderi (2008)[35] | 1 | 1 | 2 | 0 | 2 | 1 | 7 |
| Ochs-Balcom(2008)[22] | 2 | 1 | 2 | 0 | 2 | 2 | 9 |
| Theodoratou (2008)[23] | 2 | 1 | 2 | 0 | 2 | 2 | 9 |
| Jenab (2009)[12] | 1 | 2 | 2 | 1 | 2 | 2 | 10 |
| Hughes (2011)[36] | 1 | 1 | 2 | 0 | 2 | 1 | 7 |
| Mahmoudi (2011)[24] | 1 | 1 | 2 | 0 | 2 | 1 | 7 |
| Pei (2011)[13] | 1 | 2 | 2 | 0 | 2 | 1 | 8 |
| Bentley (2012)[25] | 0 | 1 | 2 | 0 | 2 | 1 | 6 |
| Yamaji (2012)[32] | 1 | 1 | 2 | 1 | 2 | 2 | 9 |
| Rasool (2013)[26] | 1 | 1 | 2 | 0 | 2 | 1 | 7 |
| Laczmanska (2014)[27] | 1 | 1 | 2 | 0 | 2 | 1 | 7 |
| Sarkissyan (2014)[28] | 1 | 1 | 2 | 0 | 2 | 1 | 7 |
| Takeshige (2015)[29] | 2 | 1 | 2 | 0 | 2 | 2 | 9 |
| Xia (2015)[37] | 1 | 1 | 2 | 0 | 2 | 1 | 7 |
| Alkhayal (2016)[30] | 1 | 2 | 2 | 0 | 2 | 1 | 8 |
| Beckett (2016)[33] | 1 | 1 | 2 | 0 | 2 | 2 | 8 |
| Xia (2016)[39] | 1 | 1 | 2 | 0 | 2 | 1 | 7 |
| Cho (2017, current) | 1 | 1 | 2 | 0 | 2 | 2 | 8 |
| Zheng (2017)[38] | 1 | 1 | 2 | 0 | 2 | 1 | 7 |

**Supplementary Figure S1. Flowchart depicting the literature search and selection process**. Three articles investigated both ulcerative colitis and Crohn's disease.

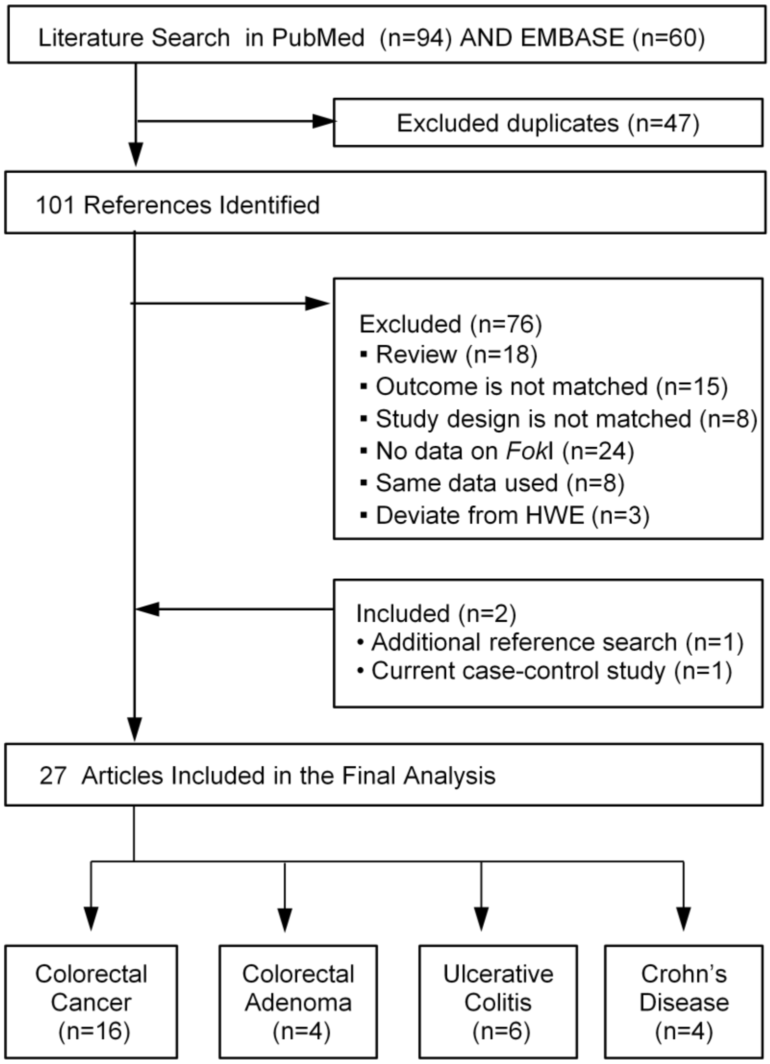

**Supplementary Figure S2. Flow diagram showing the selection of cases and controls.**

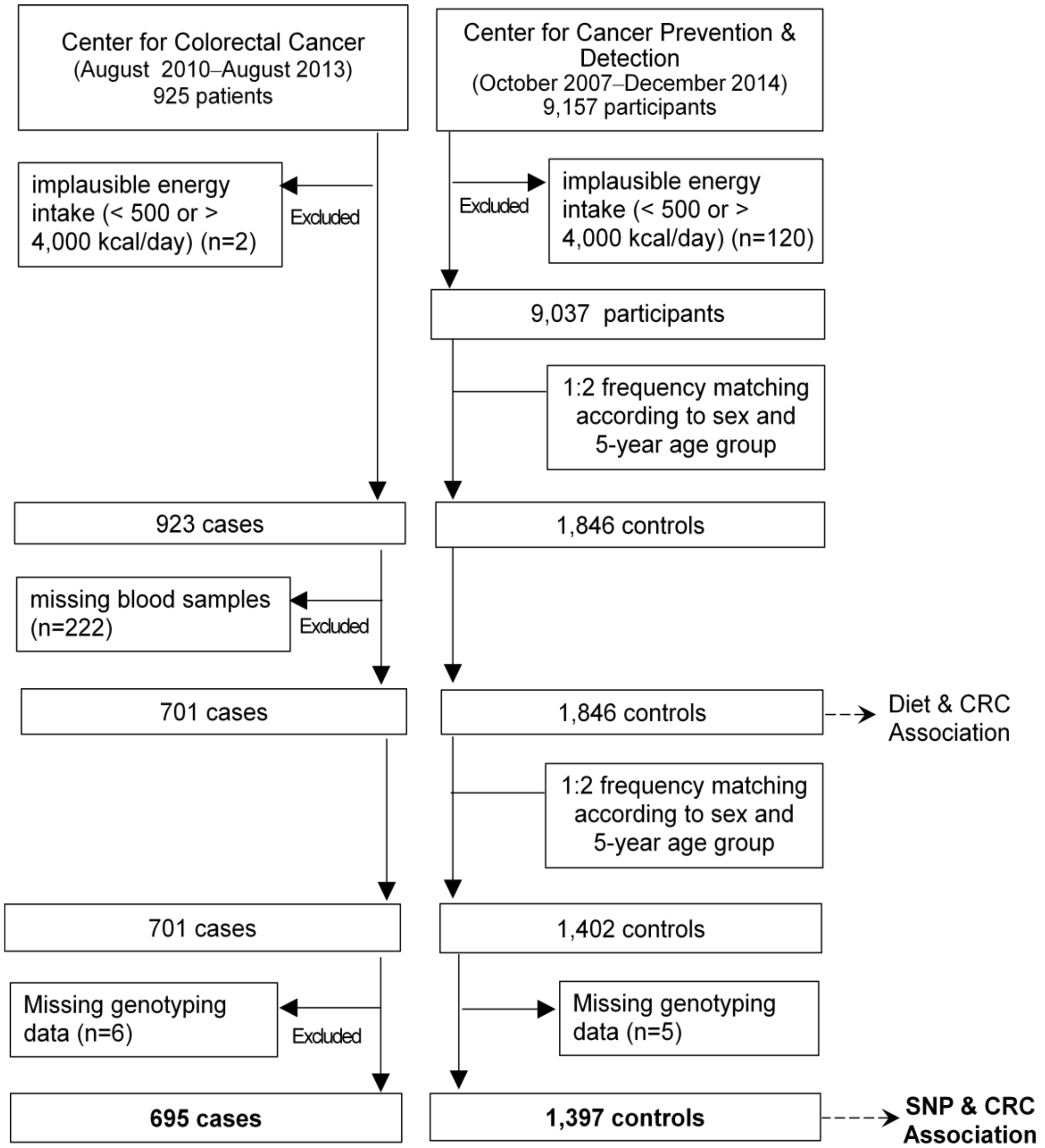

Supplement: Supplementary file 1 — Supplementary Material [file 41598_2018_31244_MOESM1_ESM.pdf]
